# Effects of High Glucose Concentration on Pericyte-Like Differentiated Human Adipose-Derived Mesenchymal Stem Cells

**DOI:** 10.3390/ijms22094604

**Published:** 2021-04-27

**Authors:** Giuliana Mannino, Anna Longo, Florinda Gennuso, Carmelina Daniela Anfuso, Gabriella Lupo, Giovanni Giurdanella, Rosario Giuffrida, Debora Lo Furno

**Affiliations:** Department of Biomedical and Biotechnological Sciences, University of Catania, 95123 Catania, Italy; giuliana.mannino@unict.it (G.M.); longo.anna@hotmail.it (A.L.); florigennuso@gmail.com (F.G.); daniela.anfuso@unict.it (C.D.A.); giuffros@unict.it (R.G.); lofurno@unict.it (D.L.F.)

**Keywords:** human adipose mesenchymal stem cells, high glucose, pericyte-like differentiation, eye diseases, diabetic retinopathy, regenerative medicine

## Abstract

A pericyte-like differentiation of human adipose-derived mesenchymal stem cells (ASCs) was tested in in vitro experiments for possible therapeutic applications in cases of diabetic retinopathy (DR) to replace irreversibly lost pericytes. For this purpose, pericyte-like ASCs were obtained after their growth in a specific pericyte medium. They were then cultured in high glucose conditions to mimic the altered microenvironment of a diabetic eye. Several parameters were monitored, especially those particularly affected by disease progression: cell proliferation, viability and migration ability; reactive oxygen species (ROS) production; inflammation-related cytokines and angiogenic factors. Overall, encouraging results were obtained. In fact, even after glucose addition, ASCs pre-cultured in the pericyte medium (pmASCs) showed high proliferation rate, viability and migration ability. A considerable increase in mRNA expression levels of the anti-inflammatory cytokines transforming growth factor-β1 (TGF-β1) and interleukin-10 (IL-10) was observed, associated with reduction in ROS production, and mRNA expression of pro-inflammatory cytokines interleukin-1β (IL-1β) and tumor necrosis factor-α (TNF-α), and angiogenic factors. Finally, a pmASC-induced better organization of tube-like formation by retinal endothelial cells was observed in three-dimensional co-culture. The pericyte-like ASCs obtained in these experiments represent a valuable tool for the treatment of retinal damages occurring in diabetic patients.

## 1. Introduction

Multipotent differentiation ability of human adipose-derived mesenchymal stem cells (ASCs) has been extensively studied in the last few decades for their potential applications in stem-cell-based therapeutic strategies. Indeed, they are able to differentiate not only into cells of mesodermal origin such as adipocytes [1,2], chondrocytes [3] and osteocytes [4], but also into cells of different lineages [5]. For this reason, they have been extensively investigated for potential therapeutic applications in a variety of pathologies, being also capable of releasing immunosuppressive cytokines such as interleukin-10 (IL-10), or factors promoting tissue regeneration (hepatocyte growth factor, and transforming growth factor-β; TGF-β) [6,7,8,9]. Stem-cell-based therapies are currently explored for the treatment of ocular diseases [10,11,12,13], including diabetic retinopathy (DR), in which a vascular complication leads to the disruption of the blood retinal barrier (BRB) [14,15,16]. Microcirculation damage is mainly ascribed to the irreversible loss of pericytes, an early hallmark of DR, because in the adult retina they are not able to replicate [17,18]. Hyperglycemia-induced overproduction of reactive oxygen species (ROS) stimulates inflammatory processes leading to retinal vasculature damages. In fact, increased levels of different inflammatory cytokines, such as interleukin-1β (IL-1β) and tumor necrosis factor-α (TNF-α) have been found in DR patients [19]. Activated microglia, endothelial cells and macroglia participate in the secretion of these cytokines, whose accumulation contributes to neuronal death [20]. Moreover, proangiogenic factors such as vascular endothelial growth factor (VEGF) and Angiopoietin-2, have been shown to induce endothelial cell proliferation, eventually leading to increased vessel permeability and breakdown [21,22].

To date, only limited positive outcomes have been obtained by using available therapeutic strategies, which are limited to the late stages of the disease, when irreversible damage has already occurred [23,24]. Therefore, a stem-cell-based therapy might represent a valuable tool to counteract the pericyte loss and slow down the progression of the disease. Encouraging results have been reported in experiments in vitro and in a murine model of DR [14]. By intravitreal injection of native or pretreated ASCs, retinal capillary dropout was prevented and the revascularization of the central retina was observed. In our previous in vitro investigation, a pericyte-like differentiation of ASCs was achieved by growing ASCs in a culture medium specifically designed for pericytes (PM) [25]. As a result, overexpression of smooth muscle actin α (α-SMA) and neural/glial antigen 2 (NG2) was obtained, as well as their typical peritubular localization. In addition, co-cultures of pericyte-like differentiated ASCs and human retinal endothelial cells (HRECs) induced an increased expression of junction proteins. From here on, they will also be called pmASCs because their differentiation process was achieved by using PM.

The purpose of the present investigation was to further explore the behavior of these pmASCs obtained by the same differentiation protocol. In particular, to mimic conditions occurring in diabetic patients, various parameters were measured and compared after the addition of glucose to the culture medium. Cell proliferation, cell viability and their migratory ability were evaluated. In addition, ROS production was measured as well as the mRNA expression of pro-inflammatory (TNF-α and IL-1β) and anti-inflammatory (TGF-β1 and IL-10) cytokines. Finally, mRNa expression of proangiogenic factors (VEGF, Angiopoietin-2 and matrix metallopeptidase-9; MMP-9) was also detected. In later experiments, three-dimensional co-cultures in Matrigel were designed to evaluate pmASC effects on HREC ability to organize tube-like formations.

Results obtained are encouraging because, even in the presence of high glucose concentrations, pericyte-like ASCs feature remarkable levels of viability, high proliferation rate and noteworthy migratory ability. Moreover, a reduced production of ROS, pro-inflammatory cytokines and pro-angiogenic factors was observed, in combination with an increased production of anti-inflammatory cytokines. Finally, positive effects were observed for HREC tube-like formations.

## 2. Results

### 2.1. Cell Proliferation and Viability

**Figure 1:**
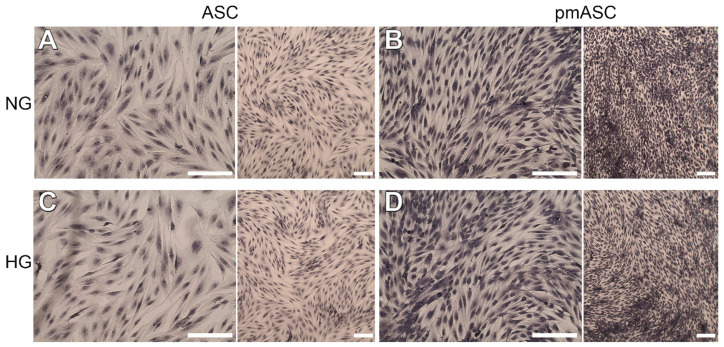
Representative photomicrographs of human adipose mesenchymal stem cells cultured in basal medium (ASC) or in pericyte medium (pmASC), stained by crystal violet during cellular proliferation assays. After three days of growth in their respective medium, glucose was added in some samples (High Glucose, 25 mM, HG), whereas other samples were kept in normal glucose (NG) condition. Pictures were taken after 72 h from glucose addition and compared to NG samples. **A**: ASC NG; **B**: pmASC NG; **C**: ASC HG; **D**: pmASC HG. In each image, two magnification levels are shown (10× and 4×, respectively). Scale bar: 100 µm.

**Figure 2:**
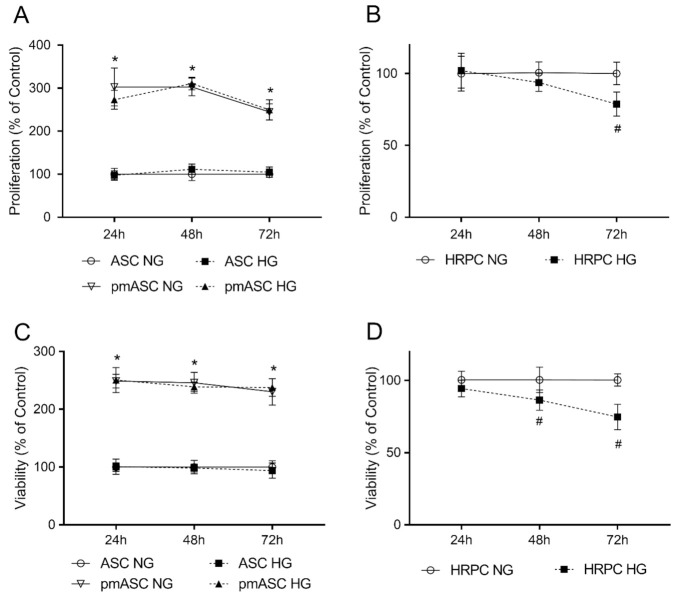
High glucose effects on cell proliferation and viability in human adipose mesenchymal stem cells cultured in basal medium (ASC) or in pericyte medium (pmASC), and in human retinal pericytes (HRPC). In some samples, glucose was added to the culture medium (High Glucose, 25 mM, HG); data were gathered after 24 h, 48 h and 72 h from glucose addition and compared to corresponding samples kept in normal glucose (NG) condition. Proliferation rate was assessed by crystal violet assays in ASC, pmASC (**A**), and HRPC cultures (**B**) in NG or in HG. Cell viability was evaluated by MTT assays in ASC, pmASC (**C**), and HRPC cultures (**D**) in NG or in HG. Absorbance values were determined at 570 nm for both assays. Values are expressed as mean ± SEM of three independent experiments. In A and C, values are referred to ASC NG population, at each corresponding time point. In B and D, values are referred to HRPC NG population, at each corresponding time point. * *p* < 0.05 pmASC vs. ASC; # *p* < 0.05 HG vs. NG. Two-way ANOVA, followed by Sidak’s test.

A high proliferation rate was observed in all ASC populations tested, although with significant differences. Figure 1 shows the typical fibroblast-like morphology in all the four subgroups studied. It can be easily noted that much denser populations are present in pmASC cultures (Figure 1B,D), regardless of different glucose concentrations. Quantitative data in Figure 2A indicate that pmASCs were more numerous by about 200%. A slight decline could be noted at longer times, likely because of high cell density. No significant differences were induced in these populations by glucose addition. Instead, a high glucose concentration negatively affected the proliferation rate of human retinal pericytes (HRPCs), which gradually decreased, especially at 72 h (−30%) from glucose addition (Figure 2B). Cell viability measurements quantified by MTT assays (Figure 2C,D) largely confirmed cell proliferation data. Similar high values were shown by pmASCs vs. ASCs, at any glucose concentration at every time point (Figure 2C). On the contrary, lower values were measured in the HRPC population (Figure 2D) after glucose addition; significant decreases were progressively observed after 48 h (−20%) and 72 h (−30%).

### 2.2. Cell Migration

**Figure 3:**
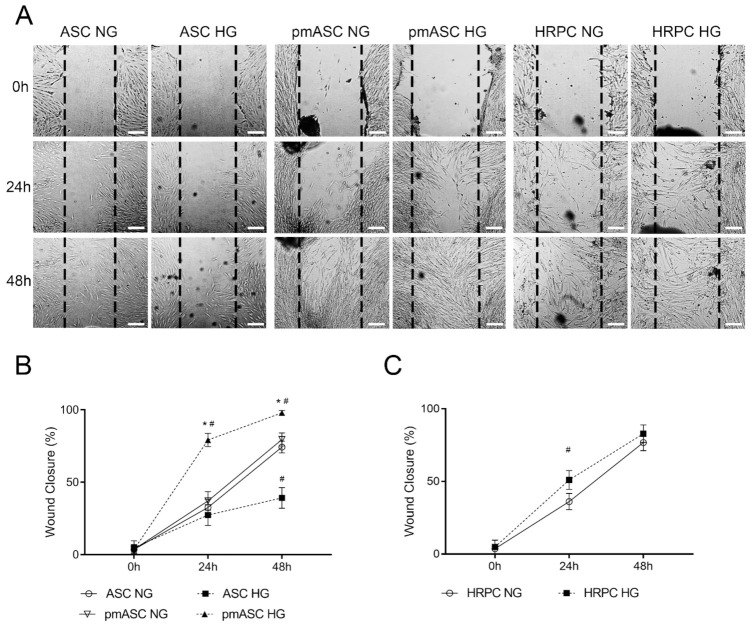
Cell migration ability evaluated by wound-healing assays in human adipose mesenchymal stem cells cultured in basal medium (ASC) or in pericyte medium (pmASC), and in cultures of human retinal pericytes (HRPC). In some samples of each culture type, glucose was added to the culture medium (High Glucose, 25 mM, HG) whereas other samples were kept in normal glucose (NG) condition. (**A**): Representative pictures of each sample taken immediately after the scratch (0 h), after 24 h and 48 h of culture. Scale bar: 100µm (Magnification: 4×). Percentage of wound closure was quantified by Image J software for ASC and pm ASC cultures (**B**), and HRPC cultures (**C**). Values are expressed as mean ± SEM of three independent experiments. * *p* < 0.05 pmASC vs. ASC; # *p* < 0.05 HG vs. NG. Two-way ANOVA, followed by Sidak’s test.

A wound-healing assay was performed to evaluate cell migration ability of the different ASC subgroups, compared to HRPCs (Figure 3A). Quantitative data show that a remarkable migratory ability was exhibited by both ASCs and pmASCs over a 48-h observation period, leading to about 70% wound closure, when cultured in normal glucose (NG) condition (Figure 3B). Significant differences were observed when glucose was added to the culture medium (HG); in fact, an opposite trend was clearly shown by ASCs (showing a wound closure of about 35%) compared to pmASCs, which virtually closed the gap. Intermediate values were observed for HRPCs with approximately 70% wound closure (Figure 3C). The relevant cell migration ability of pmASCs in HG condition was particularly evident even at 48 h from the scratch.

### 2.3. ROS Level Measurements

**Figure 4:**
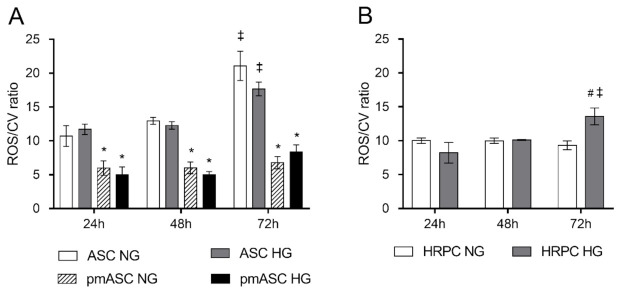
High glucose effects on ROS levels evaluated by H2DCFDA assays in (**A**) human adipose mesenchymal stem cells cultured in basal medium (ASC) or in pericyte medium (pmASC), and in (**B**) human retinal pericytes (HRPC). In some samples, glucose was added to the culture medium (High Glucose, 25 mM, HG); data were gathered after 24 h, 48 h and 72 h from glucose addition, and compared to samples kept in normal glucose (NG) condition. ROS values of each group are normalized by the corresponding population density, as determined by crystal violet staining, at each corresponding time point (ROS/CV ratio). Values are expressed as mean ± SEM of three independent experiments. * *p* < 0.05 pmASC vs. ASC; # *p* < 0.05 HG vs. NG; ‡ *p* < 0.05 72 h vs. 24 h; Two-way ANOVA, followed by Sidak’s test.

To evaluate oxidative stress, ROS levels in each ASC subgroup (Figure 4A) and HRPC population (Figure 4B) were measured and normalized by their own population density. A progressive increase of ROS levels was noted in the ASC population over the observation period, whereas steady, significantly lower values were detected for pmASCs at each corresponding time point. No evident changes were observed within each ASC group after glucose addition. A different trend was noted in HRPC cultures, where a significant higher ROS production was observed after 72 h from glucose addition.

### 2.4. High Glucose Effects on ASC mRNA Levels of Anti-Inflammatory Cytokines, Pro-Inflammatory Cytokines, and Angiogenic Factors

**Figure 5:**
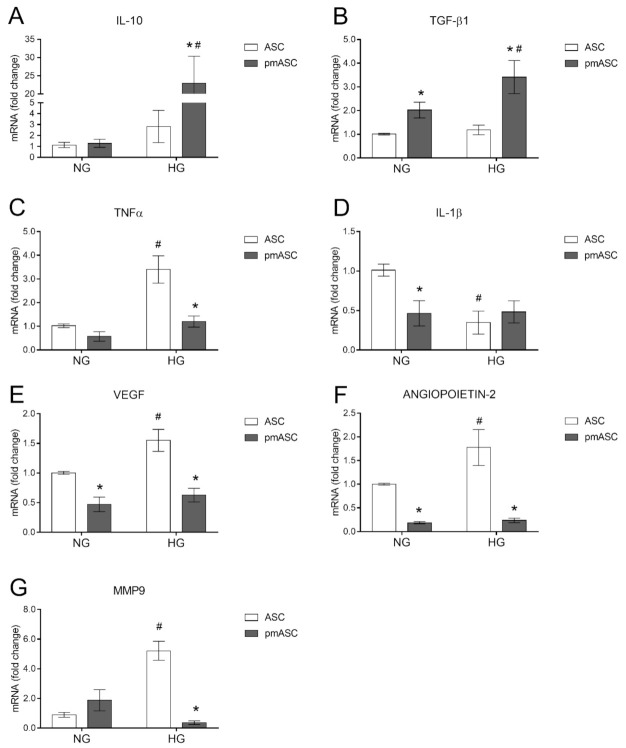
High glucose effects on anti- and pro-inflammatory cytokines and angiogenic factors in human adipose mesenchymal stem cells cultured in basal medium (ASC) or in pericyte medium (pmASC). In some samples, glucose was added to the culture medium (High Glucose, 25 mM, HG); data were gathered after 72 h from glucose addition and compared to corresponding samples kept in normal glucose (NG) condition. Quantitative analysis of IL 10 (**A**), TGF-β1 (**B**), TNFα (**C**), IL-1β (**D**), VEGF (**E**), Angiopoietin-2 (**F**) and MMP9 (**G**) mRNA levels, as evaluated by qRT-PCR. mRNA levels of each group were normalized to the housekeeping reference gene ribosomal 18S RNA. In the histograms, values are expressed as a fold change of those detected in ASCs cultured in NG condition. Each value represents mean ± SEM obtained from three independent experiments. * *p* < 0.05 pmASC vs. ASC; # *p* < 0.05 HG vs. NG. Two-way ANOVA, followed by Sidak’s test.

Inflammation-related cytokine mRNA levels were measured by quantitative RT-PCR analyses in ASC and pmASC cultures, both in NG and in HG conditions (Figure 5). No evident differences were observed for anti-inflammatory cytokine IL-10 mRNA expression between ASCs and pmASCs when cultured in NG conditions. In HG conditions, instead, IL-10 mRNA levels, slightly higher in ASC cultures, were increased 25-fold in pmASCs. Moreover, TGF-β1 mRNA levels were increased in pmASCs vs. ASC cultures, especially in HG conditions. Similar values of TNF-α mRNA levels were detected in ASC and pmASC cultures in NG conditions. In HG conditions, only weakly increased levels were observed in pmASC, compared with more evident increases in ASCs. IL-1β mRNA levels in NG conditions were significantly lower in pmASCs vs. ASCs. In HG cultures, lower levels were measured in ASCs, whereas no significant differences were noticeable in pmASCs.

In further experiments, mRNA expression levels of angiogenic factors VEGF, angiopoietin-2 and MMP9 were evaluated. Both VEGF and angiopoietin-2 values increased in ASC cultured in HG, whereas lower levels in pmASCs were not significantly affected. Dynamic effects were observed for MMP9 mRNA levels in the different conditions. In particular, low levels detected in NG conditions were significantly higher in ASCs and even lower in pmASCs following glucose addition. Overall, these data indicate a modulation of inflammation genes in favor of an anti-inflammatory phenotype. At the same time, a general decrease of angiogenic factors was observed.

### 2.5. Three-Dimensional Cultures in Matrigel

**Figure 6:**
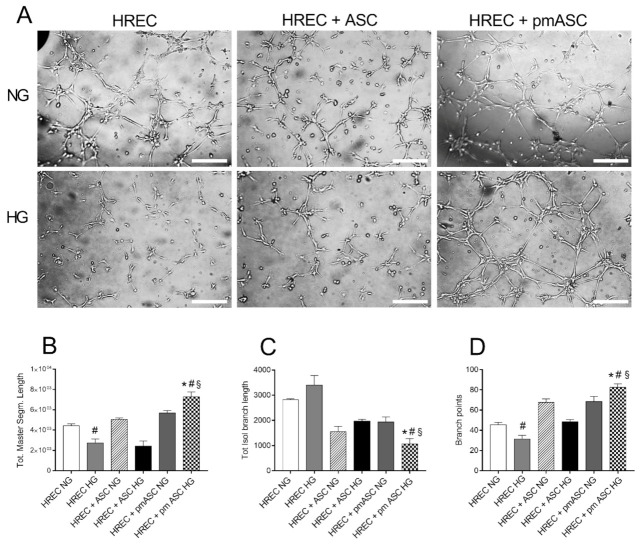
High glucose effects on tube-like structure formation by human retinal endothelial cells (HREC), or co-cultured with ASCs (HREC + ASC) or with pmASCs (HREC + pmASC). In some samples, glucose was added to the culture medium (High Glucose, 25 mM, HG) and compared to samples kept in normal glucose (NG) condition. (**A**), Representative microphotographs showing three-dimensional cultures in Matrigel of each sample. Pictures were taken at 6 h after seeding. Scale bar: 250 µm (Magnification: 4×). Histograms at the bottom show the “total master segment length” (**B**), the “total isolated branch length” (**C**) and “branching points” (**D**). Quantitative analysis was carried out using Image J software. Values are expressed as a mean ± SEM of three independent experiments. * *p* < 0.05 pmASC vs. ASC; # *p*< 0.05 HG vs. NG; § *p*< 0.05 vs. HREC HG. Two-way ANOVA, followed by Sidak’s test.

Interactions between HRECs and ASCs or pmASCs were tested in three-dimensional co-cultures, analyzing modifications induced on spontaneous HREC-assembled tube-like formations (Figure 6A). Quantitative data show that, in HG vs. NG conditions, total master segment length values (Figure 6B) were lower for HRECs, also when co-cultured with ASCs; higher values were instead measured for HRECs co-cultured with pmASCs. An opposite trend was noticed for the total isolated branch length (Figure 6C) which, in HG vs. NG conditions, produced higher values for HRECs and HRECs + ASCs and significantly lower values for HRECs + pmASCs. Branch point measurements shown in Figure 6D fit quite well with the master segment and isolated branch length values. After glucose addition, branch point numbers were lower in HRECs and in co-cultures of HRECs and ASCs, and higher in co-cultures of HRECs and pmASCs. As was expected, the number of branch points was proportionally higher with increased total master segment lengths, and inversely related to the total isolated branch length.

## 3. Discussion

Diabetes is a complex metabolic disorder, characterized by chronic hyperglycemia along with dyslipidemia, hypoinsulinemia and hypertension [20]. Hyperglycemia triggers a series of events that include inflammation, mitochondrial overproduction of ROS, neoangiogenesis and BRB breakdown. In particular, the integrity of the retinal microvasculature mainly relies on a strong pericyte coverage, which is considerably higher than in any other tissue. In fact, it is widely accepted that pericyte loss is a major cause of microvasculature damage, as it occurs in DR patients. A weakened capillary wall causes exudates, hemorrhage, retinal edema, and capillary micro-aneurysms, eventually leading to visual loss [26]. In this context, a cell-based therapeutic approach would be advisable to replace lost pericytes and restore BRB integrity. Indeed, beneficial effects were reported in rodent models of DR following intravitreal injections of native or TGF-β1 pretreated ASCs [14]. Furthermore, in a previous investigation we were able to induce a pericyte-like differentiation by growing ASCs in a culture medium specifically designed for pericytes [25]. In our opinion, this strategy offers the advantage of obtaining pericyte-like ASCs still in the proliferative stage, more suitable to restore a functional BRB. It is a pivotal issue to investigate to what extent these cells are influenced by the altered microenvironment existing in diabetic eyes, where hyperglycemia induces increased oxidative stress, inflammation, aberrant angiogenesis, and edema. Therefore, several parameters related to hyperglycemia-induced alterations were examined, such as cell proliferation, viability and migration ability, ROS production, as well as the expression of inflammation-related cytokines and angiogenic factors.

Results show that a significantly higher proliferation rate and cell viability were exhibited by pmASCs, probably due to the presence of supplementary growth factors in the pericyte medium. Instead, a progressive decline in both proliferation rate and cell viability was detected for HRPCs, starting at 48 h after glucose addition to the culture medium, as also previously reported [15]. Further positive effects can be deduced observing cell migration ability of the different populations. While ASC wound-healing activity was impaired by HG conditions, a better performance was shown by pmASCs in both NG and HG conditions, even better than HRPCs, featuring intermediate ability. Overall, it can be concluded that a hyperglycemic environment does not significantly affect pmASCs. This substantial resistance to hyperglycemia was also supported by the lower ROS production exhibited by pmASCs, if compared to native ASCs or HRPCs. This is particularly interesting because increased ROS levels are involved in the up-regulation of pro-inflammatory cytokine production [27]. Like other cells such as fibroblasts, ASCs feature a remarkable immunomodulatory action [28,29]. In the present work, ASC immunomodulatory activity was enhanced by PM treatment, as indicated by the modulation of inflammatory-related cytokine mRNA expressions. Basal values of anti-inflammatory IL-10 observed in NG conditions were remarkably increased in pmASCs, especially in HG conditions. This finding might be of crucial importance considering that in DR patients, low levels of this anti-inflammatory cytokine are related to the increased severity of the disease [30]. In addition, in vitro studies suggest that IL-10 exerts a protective role on retinal endothelial cells [31,32,33,34]. Although to a lesser extent, TGF-β1 was also increased. Among inflammatory regulatory cytokines, TGF- β exhibits important immunomodulatory and fibrogenic activities, especially during inflammation and remodeling processes [35]. It is also known that, together with IL-10, it is able to suppress several pro-inflammatory cytokines, [36] and in mouse models of retinitis pigmentosa, application of TGF-β1 was able to protect degenerating cones and save vision [37]. As a result, it can be hypothesized that inflammation-related damage would be efficiently counteracted by pmASCs, also considering that pro-inflammatory cytokines such as TNF-α and IL-1β are kept at low levels.

In DR patients, increased levels of intravitreal pro-inflammatory cytokines stimulate aberrant angiogenesis, being associated with a high production of angiogenic factors such as VEGF, angiopoietin-2 and MMP9 [20,38]. Increased levels of VEGF and angiopoietin-2 promote both increased angiogenesis and vessel leakage. In response to increased oxidative stress, MMP9 increases vascular permeability by disrupting the tight junction complex [39,40]. Indeed, in the present experiments, an increased mRNA expression of VEGF, Angiopoietin-2 and MMP9, was observed in ASC cultures after glucose addition. However, basal lower levels detected in pmASCs were virtually unaffected (VEGF and Angiopoietin-2), or further reduced (MMP9) by glucose addition. In this respect, corroborating results were obtained in three-dimensional cultures in Matrigel, examining HREC-assembled tube-like formations. In agreement with Fiori et al. (2020), when HRECs were cultured alone, high glucose concentrations induced a reduction of master segment lengths and the number of branch points; such a disarranged pattern was also suggested by the increased number of isolated branches. No significant improvements were observed when HRECs were co-cultured with ASCs. On the contrary, co-culturing HRECs with pmASCs, master segment lengths and the number of branch points were improved, whereas the number of isolated branches decreased. This might be indicative that, also in high glucose conditions, the presence of pericyte-like ASCs would induce a better organized vascular network.

In conclusion, data reported in this work indicate that a specific upstream treatment can contribute to the optimization of a stem-cell-based therapeutic application. It remains to be verified whether these encouraging results obtained in vitro really provide beneficial effects when these pericyte-like ASCs are implanted in vivo, for example by intravitreal injections in animal models of DR.

## 4. Materials and Methods

### 4.1. Materials

Culture media and supplements were purchased from Innoprot (Elexalde Derio, Spain) or Lonza (Basel, Switzerland). T25 flasks and multiwell culture plates were purchased from Corning (New York, NY, USA). MTT assay was purchased from Chemicon (Temecula, CA, USA). Cellular Reactive Oxygen Species Detection Assay Kit was purchased from Abcam (Cambridge, UK).

### 4.2. Cell Cultures

#### 4.2.1. HRPC Cultures

Primary human retinal pericytes (HRPCs) were purchased from Innoprot (Elexalde Derio, Spain). HRPCs were seeded in poly-L-lysine (0.01 mg/mL solution, Innoprot) coated culture flasks and incubated at 37 °C with 5% CO_2_ in a specific pericyte medium (PM), containing 2% fetal bovine serum (FBS), 1% pericyte growth supplement (PGS), and 1% penicillin/streptomycin (Innoprot). At about 70% confluence, the cells were trypsinized and plated for comparison with ASCs cultured in the different experimental conditions. For experimental purposes, some samples were cultured in a high glucose concentration (25 mM; HG) and compared to 5.55 mM glucose (NG) cultured cells. At 25 mM glucose concentration, the literature data report pericyte apoptosis [41,42] and altered HREC tube formation [43].

#### 4.2.2. HREC Cultures

HRECs were purchased from Innoprot and seeded in culture flasks with endothelial cell medium (ECM) (Innoprot), containing 5% FBS, 1% endothelial cell supplement factor, and 1% penicillin/streptomycin (Innoprot). Cells were incubated at 37 °C with 5% CO_2_ and, when reaching approximately 70% confluence, were trypsinized and used for co-culture experiments. For experimental purpose, some samples were cultured in HG conditions and compared to NG cultured cells.

#### 4.2.3. ASC Cultures

Adipose tissue was harvested from 4 healthy female donors (32–38 years old) undergoing liposuction procedures at the Cannizzaro Hospital, Catania (Italy). The donors were nonsmokers and were not on estrogen replacement therapy. The lipoaspirate was obtained from the abdominal region after donors signed an informed consent to use the lipoaspirate for experimental procedures, in accordance with the Declaration of Helsinki. The protocol was approved by the local ethics committee (Comitato etico Catania1; Authorization n. 155/2018/PO). The raw lipoaspirate (50–100 mL) was washed with sterile phosphate-buffered saline (PBS; Invitrogen, Monza, Italy) to remove red blood cells and debris, and incubated for 3 h at 37 °C with an equal volume of serum-free Dulbecco’s Modified Eagle’s Medium containing 5.55 mM glucose (DMEM; Sigma-Aldrich, Milan, Italy) and 0.075% type I collagenase (Invitrogen). After inactivation of collagenase activity by adding an equal volume of DMEM containing 10% FBS (Gibco, Monza, Italy), the digested lipoaspirate was centrifuged at 1200 rpm for 10 min. The pellets were then resuspended in PBS and filtered through a 100 µm nylon cell strainer (Falcon BD Biosciences, Milan, Italy). After a further centrifugation (1200 rpm for 10 min), cells were plated in T75 culture flasks (Falcon BD Biosciences) with DMEM containing 10% FBS, 1% penicillin/streptomycin, and 1% MSC growth supplement (ScienCell Research Laboratories, Milan, Italy). After 24 h incubation at 37 °C with 5% CO_2_, non-adherent cells were removed by replacing the growth medium. After reaching confluence (about 80% of total flask surface), all cultures were trypsinized and, after resuspension, cells were expanded for 3 passages before subsequent experimental procedures.

The MSC nature of ASCs used in the present study was verified in previous works, where cells of the same stock were investigated [25]. In particular, they were immuno-positive for typical MSC markers (CD44, CD73, CD90 and CD105), and immunonegative for hematopoietic stem cell markers (CD14, CD34 and CD45).

#### 4.2.4. ASCs Undergoing Pericyte-Like Differentiation

For the present investigation, two groups of cultures were initially prepared: in one group ASCs were kept for 3 days in NG DMEM containing 2% FBS; in the other group, a pericyte-like differentiation was induced by culturing ASCs for 3 days in NG PM containing 2% FBS. At the third day, the medium was replaced and cells were cultured for further three days as follows: one sample of each group was maintained in the respective NG medium, another sample was cultured in HG concentrations. Therefore, four subgroups were obtained: the first served as a control (ASC NG); in the second, ASCs were cultured in high glucose DMEM (ASC HG); in the third, pre-differentiated ASCs were maintained in PM (pmASC NG); in the fourth, pre-differentiated ASCs were cultured in high glucose PM (pmASC HG). All cells were washed twice in PBS before undergoing successive experimental procedures.

### 4.3. Cell Proliferation Assay

Crystal violet staining was used to evaluate the proliferation rate of HRPCs, ASCs and pmASCs. After 1, 2 and 3 days of culture from glucose addition, both NG and HG conditions were tested. For this purpose, cells were stained with 0.5% crystal violet solution in 20% methanol for 10 min. Cells were then washed with distilled water and left to dry. After photomicrographs were acquired (Leica), crystal violet was solubilized and absorbance values were measured at 570 nm with a microplate reader (Synergy 2-BioTek). Each assay was carried out in triplicate, from three independent experiments.

### 4.4. Cell Viability Assay

After 1, 2 and 3 days of culture from glucose addition, cell viability was evaluated in both NG and HG conditions in HRPCs, ASCs and pmASCs. For this purpose, the 3-[4,5-dimethylthiazol-2-y l]-2,5-diphenyl tetrasodium bromide (MTT assay, Chemicon, Temecula, CA, USA) was added to each sample and incubated for 3 h at 37 °C. The supernatant was then removed and 100 μL Dimethyl Sulfoxide (DMSO) were used to dissolve the precipitate. Absorbance values were determined at 570 nm in a plate reader (Synergy 2-BioTek). Each assay was carried out in triplicate, from three independent experiments.

### 4.5. Wound-Healing Assay

Cell migration ability was measured using a standard wound-healing assay. After three days of growth in their respective medium, cultures of HRPCs, ASCs and pmASCs, under both NG and HG conditions, were scratched by a p200 pipet tip and monitored after 24 h and 48 h. Cell migration ability was evaluated by considering the percentage of wound closure. For this purpose, photographs from randomly selected fields were taken at each time point (0 h, 24 h and 48 h) using a phase-contrast microscope and processed using ImageJ software (NIH, Bethesda, MD, USA).

### 4.6. ROS Measurements

ROS were measured by means of the DCFDA Cellular Reactive Oxygen Species Detection Assay Kit (ab113851, Abcam Cambridge, United Kingdom), according to the manufacturer’s protocol. HRPCs, ASCs and pmASCs, under both NG and HG conditions were incubated with 25 μM 2′,7′-dichlorodihydrofluorescein diacetate (DCFDA) in buffer solution at 37 °C for 30 min. Then, DCFDA was replaced with 100 µL of medium and ROS concentration was measured by Varioskan^TM^ (λex = 495 nm, λem = 529 nm). To compare data gathered from the different samples, raw ROS values were normalized by each corresponding population density, as determined by crystal violet assay.

### 4.7. Extraction of Total RNA and Real-Time Reverse Transcriptase-Polymerase Chain Reaction (RT-PCR)

After 3 days of culture from glucose addition, in ASCs and pmASCs, under both NG and HG conditions, qRT-PCR was performed to determine mRNA levels of IL-10, TGF- β1, IL-1β, TNF-α, VEGF, Angiopoietin-2, MMP9 and 18S rRNA. Briefly, total cellular RNA was extracted from each group using TRIzol reagent (Invitrogen, Life Technologies, Carlsbad, CA, USA) according to the manufacturer’s instructions and re-dissolved in 30 μL of RNase-free water. RNA concentrations and purity were estimated by optical density at 260 and 280 nm (Nanodrop). Reverse transcription of RNA (1 µg) into first-strand cDNA was accomplished by using 200 U of Quantiscript Reverse Transcriptase in a 20 µL reaction volume with 50 ng random hexamers, 1.25 mM dNTP, 10 mM dithiothreitol, 50 mM Tris-HCl pH 8.3, 75 mM KCl, and 3 mM MgCl_2_. cDNA synthesis was carried out at 42 °C for 20 min and subsequently stopped, and the temperature raised to 95 °C for 3 min. Aliquots of cDNA (50 ng) were amplified by using iTaq Universal SYBR Green Supermix (Biorad, Milan, Italy) in a final volume of 10 μL (0.8 μM primers, 1.6 mM Mg^2+^, 1 × SYBR Green).

The results were normalized by the product of 18S ribosomal RNA (rRNA) gene expression. Primers were purchased from Eurofins Genomics Germany GmbH. Specific primer sequences, product size and gene bank accession number are reported in Table 1. Negative controls were included in each assay. Amplifications were carried out in a Quant Studio 3 Applied Biosystems, Thermo Fischer Scientific (Waltham, MA, USA). The relative mRNA variations of target genes in each experimental group were calculated using the 2^-ΔΔ CT^ method by comparing the CT value of the gene of interest to the CT value of the selected 18S rRNA gene, considered as the internal reference control gene.

### 4.8. Three-Dimensional Cultures in Matrigel

Tube-like formations by HRECs were tested in three-dimensional cultures in Matrigel Basement Membrane Matrix system (BD Discovery Labware, Bedford, MA, USA). The experimental protocol was run according to the manufacturer’s instructions. Briefly, the gel solution was thawed at 4 °C overnight, then 96-well plates were coated with 50 μL of Matrigel per well and allowed to solidify at 37 °C for 2 h. A total of 15,000 cells per well were seeded as follows: in the first group, 15,000 HRECs alone; in the second group, 10,000 HRECs and 5000 ASCs; in the third group, 10,000 HRECs and 5000 pmASCs. In one sample of each group, glucose was added to the culture medium (25 mM), obtaining six subgroups. Each experimental condition was run in triplicate. After 6 h of incubation, tube-like structures were photographed using an inverted microscope [23]. Pictures from randomly selected fields were processed using ImageJ software. In particular, the total master segment length, the total isolated branch length and the number of branch points were calculated.

### 4.9. Statistical Analysis

All results were reported as mean ± SEM. The results were analyzed using two-way ANOVA followed by Sidak’s multiple comparisons test; differences between groups were considered significant for *p*-value < 0.05.

## Figures and Tables

**Table 1:** Primer sequences used for quantitative PCR.

Gene	Sequence (5′-3′)	Amplicon (bp)	Accession Number
IL-10	Fw: GACTTTAAGGGTTACCTGGGTTG	112	NM_000572.3
	Rv: TCACATGCGCCTTGATGTCTG		
TGF-β1	Fw: CGTCTGCTGAGGCTCAAGT	74	NM_000660.7
	Rv: CGCCAGGAATTGTTGCTGTA		
TNF-α	Fw: AGCCCATGTTGTAGCAAACC	134	NM_000594.4
	Rv: TGAGGTACAGGCCCTCTGAT		
IL-1β	Fw: AGCTACGAATCTCCGACCAC	186	NM_000576.3
	Rv: CGTTATCCCATGTGTCGAAGAA		
VEGF	Fw: ATCTTCAAGCCATCCTGTGTGC	121	NM_001025366.3
	Rv: GAGGTTTGATCCGCATAATCTG		
Angiopoietin-2	Fw: CTCGAATACGATGACTCGGTG	76	NM_001386337.1
	Rv: TCATTAGCCACTGAGTGTTGTTT		
MMP9	Fw: CACTGTCCACCCCTCAGAGC	264	NM_004994.3
	Rv: GCCAACTTGTCGGCGATAAGG		
18S rRNA	Fw: TAAGTCCCTGCCCTTTGTACACA	69	NR_146119
	Rv: GATCCGAGGGCCTCACTAAAC		

## Data Availability

No additional data are available.

## Abbreviations

ASCs	human adipose-derived mesenchymal stem cells
DR	diabetic retinopathy
ROS	reactive oxygen species
PmASCs	ASCs pre-cultured in the pericyte medium
TGF-β1	transforming growth factor-β1
IL-10	interleukin-10
IL-1β	interleukin-1β
TNF-α	tumor necrosis factor-α
BRB	blood retinal barrier
VEGF	vascular endothelial growth factor
PM	pericyte medium
α-SMA	smooth muscle actin α
NG2	neural/glial antigen 2
HRECs	human retinal endothelial cells
MMP-9	matrix metallopeptidase-9
HRPCs	pericytes
NG	normal glucose
HG	high glucose
ECM	endothelial cell medium
DCFDA	2′,7′-dichlorodihydrofluorescein diacetate
MTT	3-[4,5-dimethylthiazol-2-y l]-2,5-diphenyl tetrasodium bromide

## References

[1] Lo Furno D., Graziano A.C., Avola R., Giuffrida R., Perciavalle V., Bonina F., Mannino G., Cardile V. (2016). A citrus bergamia extract decreases adipogenesis and increases lipolysis by modulating PPAR levels in mesenchymal stem cells from human adipose tissue. PPAR Res..

[2] Furno D.L., Graziano A.C.E., Caggia S., Perrotta R.E., Tarico M.S., Giuffrida R., Cardile V. (2013). Decrease of apoptosis markers during adipogenic differentiation of mesenchymal stem cells from human adipose tissue. Apoptosis.

[3] Szychlinska M.A., Calabrese G., Ravalli S., Parrinello N.L., Forte S., Castrogiovanni P., Pricoco E., Imbesi R., Castorina S., Leonardi R. (2020). Cycloastragenol as an Exogenous Enhancer of Chondrogenic Differentiation of Human Adipose-Derived Mesenchymal Stem Cells. A Morphological Study. Cells.

[4] Luo Y., Ge R., Wu H., Ding X., Song H., Ji H., Li M., Ma Y., Li S., Wang C. (2019). The osteogenic differentiation of human adipose-derived stem cells is regulated through the let-7i-3p/LEF1/β-catenin axis under cyclic strain. Stem Cell Res. Ther..

[5] Furno D.L., Mannino G., Giuffrida R., Gili E., Vancheri C., Tarico M.S., Perrotta R.E., Pellitteri R. (2018). Neural differentiation of human adipose-derived mesenchymal stem cells induced by glial cell conditioned media. J. Cell. Physiol..

[6] Kokai L.E., Marra K., Rubin J.P. (2014). Adipose stem cells: Biology and clinical applications for tissue repair and regeneration. Transl. Res..

[7] Santos A.D.L., Da Silva C.G., Barretto L.S.D.S., Franciozi C.E.D.S., Tamaoki M.J.S., De Almeida F.G., Faloppa F. (2018). Biomechanical evaluation of tendon regeneration with adipose-derived stem cell. J. Orthop. Res..

[8] Hassanshahi A., Hassanshahi M., Khabbazi S., Hosseini-Khah Z., Peymanfar Y., Ghalamkari S., Su Y., Xian C.J. (2019). Adipose-derived stem cells for wound healing. J. Cell. Physiol..

[9] Suh A., Pham A., Cress M.J., Pincelli T., TerKonda S.P., Bruce A.J., Zubair A.C., Wolfram J., Shapiro S.A. (2019). Adipose-derived cellular and cell-derived regenerative therapies in dermatology and aesthetic rejuvenation. Ageing Res. Rev..

[10] Mead B., Berry M., Logan A., Scott R.A., Leadbeater W., Scheven B.A. (2015). Stem cell treatment of degenerative eye disease. Stem Cell Res..

[11] Elshaer S.L., Evans W., Pentecost M., Lenin R., Periasamy R., Jha K.A., Alli S., Gentry J., Thomas S.M., Sohl N. (2018). Adipose stem cells and their paracrine factors are therapeutic for early retinal complications of diabetes in the Ins2Akita mouse. Stem Cell Res. Ther..

[12] Fiori A., Terlizzi V., Kremer H., Gebauer J., Hammes H.-P., Harmsen M.C., Bieback K. (2018). Mesenchymal stromal/stem cells as potential therapy in diabetic retinopathy. Immunobiol..

[13] Kremer H., Gebauer J., Elvers-Hornung S., Uhlig S., Hammes H.-P., Beltramo E., Steeb L., Harmsen M.C., Sticht C., Klueter H. (2020). Pro-angiogenic Activity Discriminates Human Adipose-Derived Stromal Cells From Retinal Pericytes: Considerations for Cell-Based Therapy of Diabetic Retinopathy. Front. Cell Dev. Biol..

[14] Mendel T.A., Clabough E.B.D., Kao D.S., Demidova-Rice T.N., Durham J.T., Zotter B.C., Seaman S.A., Cronk S.M., Rakoczy E.P., Katz A.J. (2013). Pericytes Derived from Adipose-Derived Stem Cells Protect against Retinal Vasculopathy. PLoS ONE.

[15] Giurdanella G., Anfuso C.D., Olivieri M., Lupo G., Caporarello N., Eandi C.M., Drago F., Bucolo C., Salomone S. (2015). Aflibercept, bevacizumab and ranibizumab prevent glucose-induced damage in human retinal pericytes in vitro, through a PLA2/COX-2/VEGF-A pathway. Biochem. Pharmacol..

[16] Giurdanella G., Lupo G., Gennuso F., Conti F., Furno D.L., Mannino G., Anfuso C.D., Drago F., Salomone S., Bucolo C. (2020). Activation of the VEGF-A/ERK/PLA2 Axis Mediates Early Retinal Endothelial Cell Damage Induced by High Glucose: New Insight from an In Vitro Model of Diabetic Retinopathy. Int. J. Mol. Sci..

[17] Alikhani M., Roy S., Graves D.T. (2010). FOXO1 plays an essential role in apoptosis of retinal pericytes. Mol. Vis..

[18] Beltramo E. (2013). Pericyte Loss in Diabetic Retinopathy: Mechanisms and Consequences. Curr. Med. Chem..

[19] Feng S., Yu H., Yu Y., Geng Y., Li D., Yang C., Lv Q., Lu L., Liu T., Li G. (2018). Levels of Inflammatory Cytokines IL-1β, IL-6, IL-8, IL-17A, and TNF-α in Aqueous Humour of Patients with Diabetic Retinopathy. J. Diabetes Res..

[20] Rübsam A., Parikh S., Fort P.E. (2018). Role of Inflammation in Diabetic Retinopathy. Int. J. Mol. Sci..

[21] Peters S., Cree I.A., Alexander R., Turowski P., Ockrim Z., Patel J., Boyd S.R., Joussen A.M., Ziemssen F., Hykin P.G. (2007). Angiopoietin modulation of vascular endothelial growth factor: Effects on retinal endothelial cell permeability. Cytokine.

[22] Benest A.V., Kruse K., Savant S., Thomas M., Laib A.M., Loos E.K., Fiedler U., Augustin H.G. (2013). Angiopoietin-2 Is Critical for Cytokine-Induced Vascular Leakage. PLoS ONE.

[23] Geevarghese A., Herman I.M. (2014). Pericyte-endothelial crosstalk: Implications and opportunities for advanced cellular therapies. Transl. Res..

[24] Spencer B.G., Estevez J.J., Liu E., Craig J.E., Finnie J.W. (2020). Pericytes, inflammation, and diabetic retinopathy. Inflammopharmacology.

[25] Mannino G., Gennuso F., Giurdanella G., Conti F., Drago F., Salomone S., Furno D.L., Bucolo C., Giuffrida R. (2020). Pericyte-like differentiation of human adipose-derived mesenchymal stem cells: An in vitro study. World J. Stem Cells.

[26] Cheung N., Mitchell P., Wong T.Y. (2010). Diabetic retinopathy. Lancet.

[27] Dehdashtian E., Mehrzadi S., Yousefi B., Hosseinzadeh A., Reiter R.J., Safa M., Ghaznavi H., Naseripour M. (2018). Diabetic retinopathy pathogenesis and the ameliorating effects of melatonin; involvement of autophagy, inflammation and oxidative stress. Life Sci..

[28] Vancheri C., Mastruzzo C., Trovato-Salinaro E., Gili E., Furno D.L., Pistorio M.P., Caruso M., La Rosa C., Crimi C., Failla M. (2005). Interaction between human lung fibroblasts and T-lymphocytes prevents activation of CD4+ cells. Respir. Res..

[29] Gao F., Chiu S.M., Motan D.A., Zhang Z., Chen L., Ji H.L., Tse H.F., Fu Q.L., Lian Q. (2016). Mesenchymal stem cells and im-munomodulation: Current status and future prospects. Cell Death Dis..

[30] Zhang H., Liang L., Huang R., Wu P., He L. (2020). Comparison of inflammatory cytokines levels in the aqueous humor with diabetic retinopathy. Int. Ophthalmol..

[31] Mtairag E.M., Chollet-Martin S., Oudghiri M., Laquay N., Jacob M.-P., Michel J.-B., Feldman L.J. (2001). Effects of interleukin-10 on monocyte/endothelial cell adhesion and MMP-9/TIMP-1 secretion. Cardiovasc. Res..

[32] Gimeno M.J., Pascual G., García-Honduvilla N., Prieto A., De Mon M.A., Bellón J.M., Buján J. (2003). Modulatory role of IL10 in endothelial cell damage and platelet adhesion. Histol. Histopathol..

[33] Kinzenbaw D.A., Chu Y., Silva R.A.P., Didion S.P., Faraci F.M. (2013). Interleukin-10 protects against aging-induced endothelial dysfunction. Physiol. Rep..

[34] Wang Y., Chen Q., Zhang Z., Jiang F., Meng X., Yan H. (2014). Interleukin-10 overexpression improves the function of endothelial progenitor cells stimulated with TNF-? Through the activation of the STAT3 signaling pathway. Int. J. Mol. Med..

[35] Yang Y.C., Zhang N., Van Crombruggen K., Hu G.H., Hong S.L., Bachert C. (2012). Transforming growth factor-beta1 in inflammatory airway disease: A key for understanding inflammation and remodeling. Allergy.

[36] Abdoli A., Maspi N., Ghaffarifar F. (2017). Wound healing in cutaneous leishmaniasis: A double edged sword of IL-10 and TGF-β. Comp. Immunol. Microbiol. Infect. Dis..

[37] Wang S.K., Xue Y., Cepko C.L. (2020). Microglia modulation by TGF-β1 protects cones in mouse models of retinal degeneration. J. Clin. Investig..

[38] Scholz A., Plate K.H., Reiss Y. (2015). Angiopoietin-2: A multifaceted cytokine that functions in both angiogenesis and inflammation. Ann. New York Acad. Sci..

[39] Giebel S.J., Menicucci G., McGuire P.G., Das A. (2005). Matrix metalloproteinases in early diabetic retinopathy and their role in alteration of the blood–retinal barrier. Lab. Investig..

[40] Navaratna D., McGuire P.G., Menicucci G., Das A. (2007). Proteolytic Degradation of VE-Cadherin Alters the Blood-Retinal Barrier in Diabetes. Diabetes.

[41] Devi T.S., Hosoya K.-I., Terasaki T., Singh L.P. (2013). Critical role of TXNIP in oxidative stress, DNA damage and retinal pericyte apoptosis under high glucose: Implications for diabetic retinopathy. Exp. Cell Res..

[42] May J.M., Jayagopal A., Qu Z.-C., Parker W.H. (2014). Ascorbic acid prevents high glucose-induced apoptosis in human brain pericytes. Biochem. Biophys. Res. Commun..

[43] Haspula D., Vallejos A.K., Moore T.M., Tomar N., Dash R.K., Hoffmann B.R. (2019). Influence of a Hyperglycemic Microenvironment on a Diabetic Versus Healthy Rat Vascular Endothelium Reveals Distinguishable Mechanistic and Phenotypic Responses. Front. Physiol..

